# Differential role of STIM1 and STIM2 during transient inward (*T*_in_) current generation and the maturation process in the *Xenopus* oocyte

**DOI:** 10.1186/s12899-014-0009-x

**Published:** 2014-11-15

**Authors:** Barbara Serrano-Flores, Edith Garay, Francisco G Vázquez-Cuevas, Rogelio O Arellano

**Affiliations:** Departamento de Neurobiología Celular y Molecular, Instituto de Neurobiología, Universidad Nacional Autónoma de México, Boulevard Juriquilla 3001, Juriquilla Querétaro, Querétaro, C.P. 76230 Mexico

**Keywords:** SOCE, STIM1, STIM2, *Xenopus* oocyte, Ca^2+^-entry, Maturation

## Abstract

**Background:**

The *Xenopus* oocyte is a useful cell model to study Ca^2+^ homeostasis and cell cycle regulation, two highly interrelated processes. Here, we used antisense oligonucleotides to investigate the role in the oocyte of stromal interaction molecule (STIM) proteins that are fundamental elements of the store-operated calcium-entry (SOCE) phenomenon, as they are both sensors for Ca^2+^ concentration in the intracellular reservoirs as well as activators of the membrane channels that allow Ca^2+^ influx.

**Results:**

Endogenous STIM1 and STIM2 expression was demonstrated, and their synthesis was knocked down 48–72 h after injecting oocytes with specific antisense sequences. Selective elimination of their mRNA and protein expression was confirmed by PCR and Western blot analysis, and we then evaluated the effect of their absence on two endogenous responses: the opening of SOC channels elicited by G protein-coupled receptor (GPCR)-activated Ca^2+^ release, and the process of maturation stimulated by progesterone. Activation of SOC channels was monitored electrically by measuring the *T*_in_ response, a Ca^2+^-influx-dependent Cl^−^ current, while maturation was assessed by germinal vesicle breakdown (GVBD) scoring and electrophysiology.

**Conclusions:**

It was found that STIM2, but not STIM1, was essential in both responses, and *T*_in_ currents and GVBD were strongly reduced or eliminated in cells devoid of STIM2; STIM1 knockdown had no effect on the maturation process, but it reduced the *T*_in_ response by 15 to 70%. Thus, the endogenous SOCE response in *Xenopus* oocytes depended mainly on STIM2, and its expression was necessary for entry into meiosis induced by progesterone.

**Electronic supplementary material:**

The online version of this article (doi:10.1186/s12899-014-0009-x) contains supplementary material, which is available to authorized users.

## Background

For approximately three decades, the *Xenopus* oocyte has been a useful cell model to determine the underlying mechanisms responsible for the increase of the cytoplasmic Ca^2+^ concentration through its release from intracellular reservoirs [1,2] and by calcium influx either through Ca^2+^-dependent voltage-dependent channels or via store-operated Ca^2+^ (SOC) channels [3-5]. The latter results from the activation of the phenomenon known as store-operated Ca^2+^ entry (SOCE), which allows the replenishment of emptied reservoirs [5] after the stimulation of Ca^2+^ release through IP_3_/diacylglycerol synthesis by phospholipase C (PLC). Release of Ca^2+^ from intracellular reservoirs and SOCE activation are common responses in the *Xenopus* oocytes since they endogenously express the machinery that activates PLC by stimulating endogenous G protein-coupled receptors (GPCR); cytoplasmic Ca^2+^-increase, through either release or influx, opens Ca^2+^-dependent Cl^−^ channels in the oocyte membrane generating conspicuous current responses [6]. SOCE activation in the membrane of the *Xenopus* oocyte was first detected by measuring the transient inward (*T*_in_) current response [6] after Ca^2+^ release in the oocyte. The *T*_in_ response is generated by hyperpolarizing steps, and is mainly due to the Ca^2+^-influx that subsequently opens Ca^2+^-dependent Cl^−^ channels; this membrane response has been used as a reliable monitor of SOC channel activation [3,7].

The SOCE current is most likely driven through Ca^2+^-permeable channels formed by Orai, a channel activated by association with the stromal interaction molecule (STIM) [8], a protein that is localized mainly in the endoplasmic reticulum (ER) membrane and that senses the Ca^2+^ concentration in its lumen [9]. Although transcripts for endogenous Orai and STIM molecules have been reported in the oocyte [10], the role for the different types and isoforms of these proteins and their relation with endogenous responses in the oocyte have not been thoroughly studied; these issues are of interest given that the roles for the different SOC molecular elements are also incompletely understood, and their study in a well-known model such as the *Xenopus* oocyte might reveal important information.

Two STIM proteins, STIM1 and STIM2, are expressed in eukaryotic cells [11]. A different role for each of them has been proposed; for example, the ER Ca^2+^ content must be greatly reduced in order to activate STIM1 protein, while the more Ca^2+^-sensitive STIM2 seems to require only a slight reduction in ER Ca^2+^ concentration [12-14]. It has been proposed that STIM2 participates in maintaining the cytoplasmic Ca^2+^ concentration [12-15]. Although the fundamental role of STIM1 in activating SOCE has been demonstrated in several cell types [16-18], other information indicates that STIM2 is the main protein involved in SOCE generation in neurons, dendritic cells, and mammary epithelial cells [19-21]. Thus, it is plausible that the specific functions of STIM1 and STIM2 depend on the cell type, their relative rates of expression, and other factors such as interactions among them or with regulatory proteins.

It has also been shown that during maturation, the Ca^2+^-signaling pathway in the oocyte is significantly reconfigured, probably as part of the mechanism that prepares the gamete for fertilization and subsequent embryonic development. This reconfiguration includes Orai1 channels and STIM1, which are regulated during maturation thus eliminating the SOCE response [22-25]. Due to the importance of this phenomenon for cell cycle control in general, it is also of interest to explore the effects on oocyte maturation of altered STIM expression [26,27].

In the present study, we specifically knocked down STIM1 or STIM2 in the *Xenopus* oocyte to analyze the effect on two endogenous phenomena, the generation of the *T*_in_ current response (i.e., SOC channel activation) and the maturation process. We found that STIM2 expression was essential in both phenomena, while STIM1 expression was not.

## Methods

### Cell preparation

*Xenopus laevis* frogs were obtained from Xenopus I (Ann Arbor, MI, USA). Ovary lobules [28] were surgically removed under sterile conditions from frogs that had been anaesthetized using 0.1% aminobenzoic acid ethyl ester and rendered hypothermic. After surgery, frogs were sutured and allowed to recover from anesthesia. Frogs were maintained for 3–7 days in individual tanks until healing was complete; they were then housed in larger groups, and no further oocytes were taken from them for at least 2 months. Procedures were approved by the institutional animal committees (INB-UNAM). The lobules were placed in sterile Barth’s solution containing (in mM): 88 NaCl, 1 KCl, 2.4 NaHCO_3_, 0.33 Ca(NO_3_)_2_, 0.41 CaCl_2_, 0.82 MgSO_4_, and 5 HEPES, with 75 μg/ml gentamicin and adjusted to pH 7.4. Studies were carried out using oocytes at stage VI [29] dissected from the ovaries and defolliculated by collagenase (1 mg/ml) treatment at room temperature for 30 min in normal frog Ringer’s solution (NR, containing in mM: 115 NaCl, 2 KCl, 1.8 CaCl_2_, 5 Hepes, pH 7.0). After washing, the oocytes were stored at 18°C in sterile Barth’s solution, and electrical recordings were performed over a period of 2–4 days in either uninjected oocytes or in those injected with cRNA for specific receptors and/or with antisense oligonucleotide to knock down specific proteins.

### Reverse transcription polymerase chain reactions

Total RNA from the oocytes was purified using Trizol Reagent (Life Technologies). First-strand cDNA was synthesized using 2 μg of DNase-treated RNA as template and 1 μg of oligo (dT), 0.25 μg random hexamers, and reverse transcriptase. The cDNA was used as template in a polymerase chain reaction to amplify cDNA fragments for *stim1* and *stim2,* and the ribosomal protein S2 (*rps2*) was used as a control. All the PCR programs started at 95°C for 2 min. The amplification in the 35 cycles consisted in 45 s at 95°C, 40 s at 55°C, and 35 s at 72°C, and a final extension at 72°C for 5 min. The sequences of oligonucleotides used were: *stim1*, forward, 5'-CGACGAGTTTCTCAGGGAAG-3' and reverse, 5'-CTTCATGTGGTCCTCGGAGT-3'; *stim2*, forward, 5'-CCAGCCTTGAGGCAATATGT-3' and reverse, 5'-GCAACCTCCAACTCCGATTA-3'; *rps2*, forward, 5'-TGGTAACAGGGGAGGTTTCCGC-3' and reverse, 5'-ATACCAGCCATCATGAGCAGC-3'.

The amplified products were isolated, purified (QIAEX II, QIAGEN, Hilden, Germany), and subcloned into the pJET 1.2 vector (Thermo Fisher Scientific Inc., Waltham, MA). Finally, their nucleotide sequences were confirmed by Sanger sequencing (ABI PRISM 310 Genetic Analyzer, Applied Biosystems).

### Western blot

Protein expression was assessed by Western blot in either control oocytes or in those injected with as-STIM1 or as-STIM2. For each group, 10 oocytes were homogenized 72 h post-injection in a buffer containing (in mM): 20 Tris–HCl pH 7.6, 1 EDTA pH 8, 80 sucrose, and 1X complete mini protease inhibitor (Hoffmann-La Roche, Switzerland). Then samples were centrifuged at 4°C and 500 rpm for 5 min, at 3500 rpm for 10 min, and at 14,000 rpm for 20 min. Subsequently, the final pellets were resuspended in 50 μl of buffer containing (in mM): 50 Tris–HCl pH 7.6, 1 EDTA pH 8, 100 NaCl, 100 MgCl_2_, and 1X complete mini protease inhibitor. Total membrane protein concentration was quantified with a Bradford assay. For electrophoresis, samples (1.5 μg per lane) were fractionated in a 10% SDS-polyacrylamide gel and transferred to a nitrocellulose membrane (BioRad, Hercules, CA, USA). Membranes were blocked for 1 h at room temperature in TBS-T solution (in mM): 150 NaCl, 20 Tris, pH 7.4, and 0.1% Tween 20, containing 5% nonfat dry milk and then incubated overnight at 4°C with a 1:1000 dilution of rabbit primary antibody. The antibody denoted NH-STIM1 (Alomone, Jerusalem, Israel) was directed against a region of the amino-terminus of the STIM1 protein, and the antibodies denoted NH-STIM2 (Alomone, Jerusalem, Israel) and COOH-STIM2 (ProSci Inc., Poway CA, USA) were against the amino and carboxy termini, respectively, of STIM2. Western blot analysis was also used to detect SERCA2 expression, used as a loading control (antibody from Cell Signaling Technology Inc. Danvers, MA, USA). After incubation, the membranes were washed with TBS-T and incubated for 45 min at room temperature with HRP-conjugated goat anti-rabbit antibody (Life Technologies) in TBS-T. The immunoreactive proteins were detected by chemiluminescence, and analyzed with ImageJ Software (NIH, USA); the results were normalized against the control condition and expressed in optical density units. To analyze loading controls such as SERCA2, the same membranes used to detect STIM proteins were incubated for 30 min in striping solution (in mM): 50 Tris pH 6.8, 100 β-mercaptoethanol, and 2% SDS at 55°C and then washed twice with TBS-T. Then the membranes were treated with a primary antibody against the SERCA2 protein and finally with an HRP-conjugated goat anti-rabbit antibody (Life Technologies) in TBS-T and quantified as above.

### Expression of purinergic and muscarinic receptors and transcript knockdown using antisense oligonucleotides in *Xenopus laevis* oocytes

In order to express the desired membrane receptors, cDNA coding for P2Y2, P2Y8, or M1 receptors were cloned into the plasmid pEXENEX1 and linearized with SalI or HindIII, then purified and transcribed to capped RNA with T7 polymerase using the mMESSAGE mMACHINE kit (Life Technologies CA, USA). Oocytes were injected with 25–50 ng of the respective cRNA (1 ng/nl). For purinergic receptors the P2Y8 *Xenopus laevis* [cDNA clone MGC: 52559, Source BioScience Nottingham, UK], and the P2Y2 *Xenopus tropicalis* [cDNA clone IMAGE 5383884, ATCC Manassas, USA] subtypes were used, and for muscarinic receptors, the M1 subtype [human cDNA Clone ID IOH56940 (Life Technologies CA, USA)]. Another group was injected with 25–50 nl of H_2_O for control experiments.

The antisense sequences were designed to target the initiation translation region, a strategy that has been successfully used in several experimental protocols; antisense oligonucleotide strongly inhibits mRNA expression via an RNAse-H-dependent mechanism [30].

Expression of endogenous STIM1 or STIM2 was knocked down by the injection of 25–50 ng of antisense oligonucleotides with the following sequences: for antisense oligonucleotide STIM1 (as-STIM1), 5´-ATAGCAGAGTCCGACACCAAAGCATTCCGC-3´, and for antisense oligonucleotide STIM2 (as-STIM2), 5´-TCCTCTTCTTCTTTCTCCCGTTCATGGCTG-3´. Control experiments for antisense oligonucleotides were performed injecting (50 ng per oocyte) scrambled sequences for both as-STIM, and a second control for antisense oligonuleotide injection (as-Cx38) was made knocking down the expression of connexin 38 (Cx38) which was monitored measuring the I_c_ current in Ca^2+^-free Ringer solution [28], the sequence for as-Cx38 was: 5´-GCTTTAGTAATTCCCATCCTGCCATGTTTC-3´. In general, after injection, oocytes were incubated at 18°C in Barth’s solution, and the effects of these procedures on protein expression and current responses were examined by biochemical and electrophysiological methods. Unless otherwise stated, groups of injected oocytes that were induced to express purinergic receptors were incubated in Barth’s solution containing 5 U/ml apyrase to hydrolyze the ATP that is released from the oocyte into the medium, thus avoiding stimulation of purinergic receptors during the incubation period [31].

### Electrophysiology

Oocyte membrane currents were monitored using the two-electrode voltage-clamp technique. The cells were continuously superfused (10 ml/min) with NR solution and held at −10 mV. Voltage steps to −100 mV with a duration of 4 s were applied every 40 s to activate the *T*_in_ current response, and the oocytes were stimulated for 120 s (acute protocol stimulation) with one of the agonists (100 μM ATP or ACh, or a 1:1000 dilution of FBS) added to the bath solution. For long-lasting stimulation, GPCR-expressing oocytes were incubated for 1–4 h with 1 μM agonist, and P2Y8- or P2Y2-expressing oocytes were incubated in medium devoid of apyrase; in this condition, endogenously released ATP activated the receptors in most cases.

Intra-oocyte injection of antibodies during electrophysiological recording was achieved by pneumatic pressure ejection from a third micropipette [32]. The injection micropipette was loaded with antibody dissolved in 5 mM HEPES, adjusted to pH 7.0 with KOH.

### Oocyte maturation assays

Maturation studies were carried out on batches of 15–25 defolliculated oocytes, stage VI, incubated in 2 ml of Barth’s solution plus 10 μM progesterone. GVBD was scored by white-spot formation and confirmed by cutting the oocytes through the equator after incubating them in hot NR for 1 min [32]. Maturation was analyzed in groups of oocytes that had been injected 72 h earlier with as-STIM1 or as-STIM2, and they were compared with uninjected oocytes or those injected with H_2_O. Electrical properties of oocytes from the different groups were analyzed after 9–12 h in the presence of progesterone.

### Reagents

ATP, ACh, apyrase, collagenase type I, progesterone, FBS, and all salts were from Sigma Chemical Co. (St Louis, MO, USA).

### Statistical analysis

All data are expressed as mean ± SEM of at least 10–15 oocytes from three different frogs for each condition. Statistical analysis was performed using the Igor Pro Wavemetrics, Inc. software through analysis of variance (ANOVA). The means of two different experimental groups were compared using a Student’s *t*-test. Differences were considered to be significant at p <0.01.

## Results

### Expression of endogenous STIM1 and STIM2 in *Xenopus* oocytes

**Figure 1:**
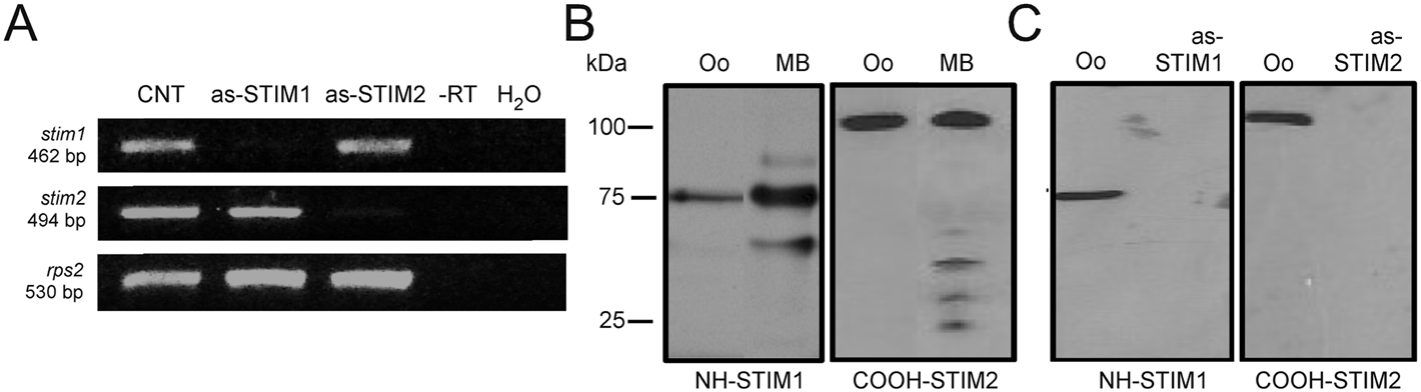
**STIM expression in the** ***Xenopus*** **oocyte and its downregulation by as-STIM injection. A)** shows the RT-PCR amplification of products that corresponded to the size expected for either *stim1* or *stim2* in native oocytes (CNT); the corresponding amplicons were absent in oocytes from the same batch that had been injected with either as-STIM1 or as-STIM2 48 h before the assay. The *rps2* amplicon indicates the reaction efficiency, and -RT and H_2_O lanes correspond to negative controls, either RNA without RT, or to the reaction mix without a cDNA template, respectively. **B)** STIM1 and STIM2 were identified by Western blot analysis in protein extracts from oocytes (Oo) or mouse brain (MB, positive control) using either NH-STIM1 (left panel) or COOH-STIM2 (right panel) as antibody. **C)** A similar analysis as in **B** was made for batches of oocytes injected with H_2_O as control (CNT), or with as-STIM1 or as-STIM2 48 h before the protein extraction, in which cases proteins were eliminated. (in all cases 10 oocytes per condition).

Expression of RNA transcripts *stim1* and *stim2* was determined in oocytes using RT-PCR. In RNA samples from control (non-injected) oocytes (Figure 1A) the use of oligonucleotide primers for *stim1* resulted in an amplicon of 463 bp, while *stim2* primers amplified a fragment of 494 bp. Both had the expected size for the corresponding transcript, and the amplified fragments were then cloned into the pJET 1.2 vector, sequenced, and analyzed using BLAST. The sequences obtained were highly homologous to those reported for *stim1* (99%) from *Xenopus laevis* [GenBank: NM_001097037.1] and for *stim2* (90%) from *Xenopus tropicalis* [GenBank: XM_004916759.1] (Additional file 1). Control amplifications without RT or without a cDNA template did not produce any PCR products (Figure 1A). Groups of oocytes that had been injected 48–72 h earlier with either the as-STIM1 or the as-STIM2 oligonucleotide sequences showed a dramatic decrease in the corresponding transcripts.

**Figure 2:**
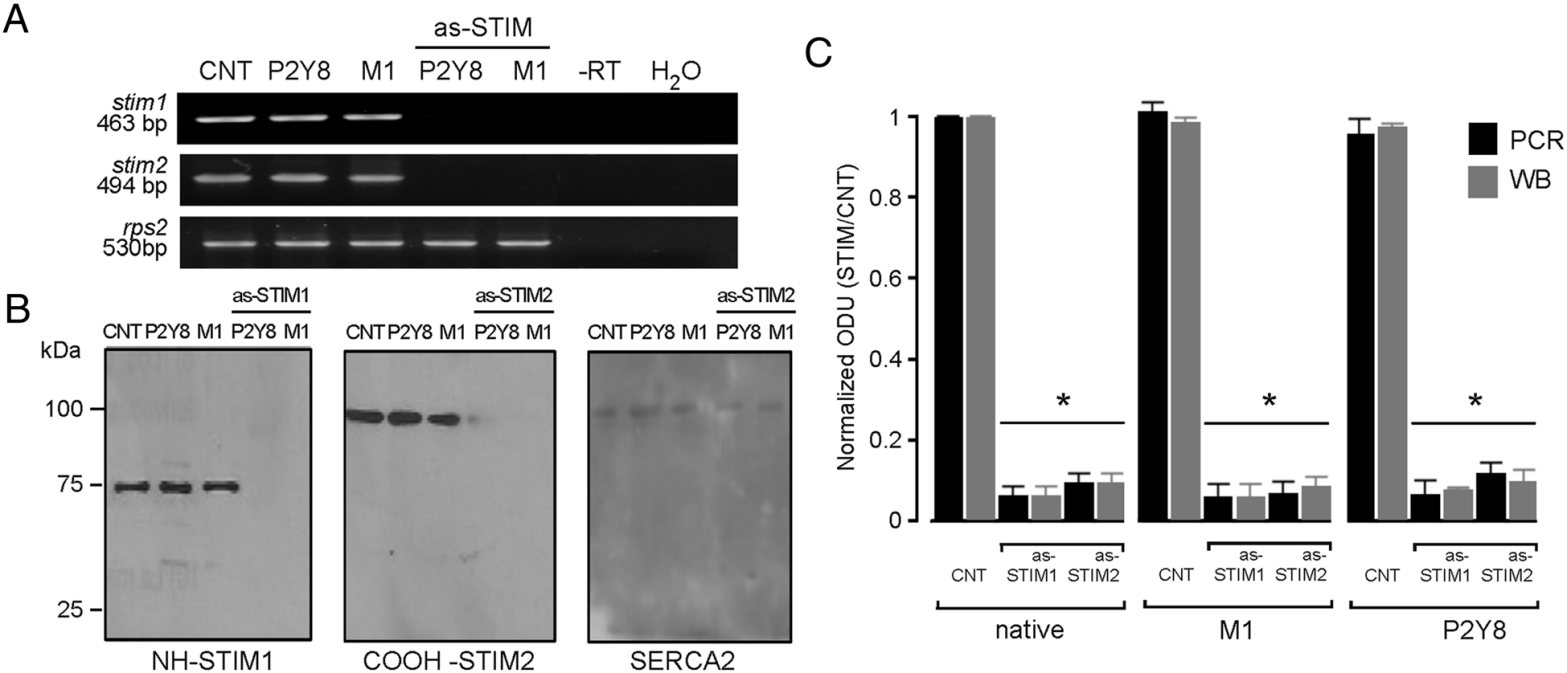
**Knockdown of STIM expression in oocytes co-injected with GPCR mRNA. A)** RT-PCR amplification of *stim1*, *stim2*, or *rps2* in batches of oocytes injected with H_2_O (CNT) or with cRNA (50 ng per oocyte) coding for either P2Y8 or M1 GPCR. In oocytes co-injected with as-STIM1 or as-STIM2 (50 ng per oocyte) together with P2Y8 or M1 cRNA, the corresponding STIM amplicon was downregulated. Control reactions illustrate specificity; *rps2* amplicons are positive controls, and -RT and H_2_O lanes show negative controls. **B)** Similar groups of oocytes as in **A)** were assayed using the Western blot technique; in this case oocytes from the same donor injected with one GPCR mRNA (P2Y8 or M1) alone, or co-injected with as-STIM1, were tested with NH-STIM1, while as-STIM2-injected oocytes were probed with COOH-STIM2. In both as-STIM groups SERCA was used as gel-loading control. **C)** The graph shows the densitometric analysis of bands, summarizing the results obtained in different preparations of 10 oocytes per group and repeated in 3–5 frogs. Both PCR products and bands detected by Western blot (WB) were analyzed for batches of oocytes injected with H_2_O (CNT) or with either 50 ng as-STIM1 or as-STIM2 alone (native group). Similar analysis was made for batches of control oocytes injected with P2Y8 or M1 cRNA alone, and oocytes from the same frogs co-injected with either as-STIM or as-STIM together with the GPCR cRNA. Optical density units (ODU) for each band were normalized against the value obtained in the corresponding CNT conditions (*p < 0.01).

To determine whether the injection of antisense oligonucleotides induced a parallel reduction in level of STIM1 and STIM2 proteins, these were evaluated using Western blot analysis with specific antibodies (Figures 1 and 2). As expected, NH-STIM1 detected a band above 75 KDa in total membrane fractions from control oocytes, and from the mouse brain (Figure 1B) [33,34]. Then, a group of oocytes injected with as-STIM1 was tested; as illustrated in Figure 1C, antisense-injected oocytes showed a significant STIM1 decrease compared to the control group.

Similarly, STIM2 was detected using two distinct antibodies, COOH-STIM2 (Figures 1B-C) and NH-STIM2, which revealed STIM2 as a band about 100 KDa, in both the total membrane preparation of control oocytes and in total protein from mouse brain, in agreement with previous reports [12,21]. Western blot analysis in oocytes injected with as-STIM2 indicated that antisense produced a large decrease in the amount of STIM2 as compared to control oocytes (Figure 1C).

These results showed that the antisense sequences used specifically decreased the endogenous transcripts for *stim1* or *stim2*, with a concomitant depletion of STIM1 and STIM2 proteins*.*

### STIM1 and STIM2 levels were decreased by injection of antisense-STIM sequences in oocytes co-expressing GPCR

Most of the following experiments were made using oocytes exogenously expressing muscarinic or purinergic receptors due to injection of the respective cRNA in order to get a robust and consistent response; therefore, it was determined if GPCR expression affected either the endogenous expression of STIM or its decrease due to as-STIM injection. Seventy-two hours after injection with cRNA coding for GPCR, oocytes exhibited strong current responses in the presence of their respective agonists (see below); this GPCR expression did not affect the level of *stim1* or *stim2* transcripts, as illustrated in Figure 2A. Moreover, the decrease of *stim1* or *stim2* expression due to antisense injection was also not affected in oocytes that were co-injected with either P2Y8 or M1 receptor cRNA (Figure 2A). Consistent with this result, knockdown of neither STIM1 nor STIM2 protein was altered by co-injecting oocytes with cRNA to express GPCR, as shown in Figure 2B. In all cases, SERCA2 (114 KDa, used as the loading control) did not show significant changes in response to the various experimental conditions (Figure 2B).

The results obtained for all conditions described above are summarized in Figure 2C. Taken together, they confirm that antisense knockdown of endogenous STIM proteins in the oocyte was specific and effective and that this was not affected by simultaneous exogenous GPCR expression.

### STIM protein knockdown did not affect the generation of the oscillatory Cl^−^ current induced by agonist

**Figure 3:**
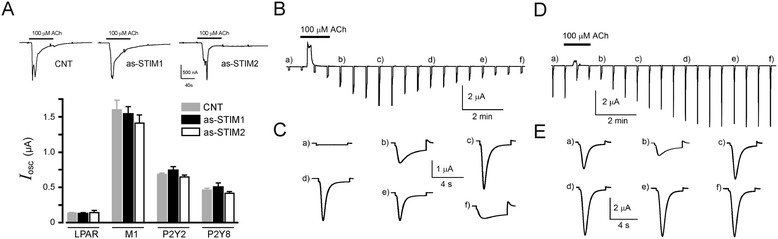
***I***_**osc**_ **and** ***T***_**in**_ **responses activated by agonist stimulation. A)** Strength of *I*_osc_ elicited by first agonist application did not change by knockdown of STIM1 or STIM2, compared with that obtained in CNT oocytes; top traces are typical responses elicited by ACh, similar responses were obtained by FBS or ATP applications, and the graph shows the average *I*_osc_ responses obtained in oocytes held at −60 mV. **B)** Record illustrating the activation of *T*_in_ current obtained in an oocyte expressing the M1 receptor by a single ACh (100 μM) application for 40 s (acute protocol). Oocytes were held at −10 mV while being superfused with NR solution and stepped to −100 mV for 4 s every 40 s; sudden hyperpolarization generated *T*_in_ current responses that follow consistent kinetics with a peak amplitude response at 280–360 s (c); after that the response was washed out with a similar time course. **C)** Shows the *T*_in_ current during the steps from −10 to −100 mV indicated with letters in panel **B)**. **D)** A similar *T*_in_ current response elicited in an oocyte from the same frog that was pre-incubated with 1 μM ACh for 4 h (long-lasting protocol), then monitored with the same electrical recording parameters and stimulated with 100 μM ACh. **E)** Shows the *T*_in_ responses indicated with the same letters as in **D)**. In this protocol *T*_in_ current was consistently activated from the beginning of the record, and a transient inhibition of the response was noted during application of the agonist (*b)*; after that, *T*_in_ recovered and remained fully activated for a long period of time. Similar responses were obtained using oocytes expressing P2Y receptors and stimulating with ATP.

To evaluate the role of STIM1 and STIM2 in SOC current activation, we monitored the *T*_in_ response generation. Prior to this analysis we tested whether or not as-STIM injection affected the Ca^2+^-dependent Cl^−^ oscillatory current (*I*_osc_) generated by the first application of one of the agonists (Figure 3A). For this, groups of oocytes were exposed to either FBS (stimulation of the endogenous LPA receptor (LPAR)) [35], ACh, or ATP in order to monitor the *I*_osc_ amplitude, as a measure of the oocyte capacity to release Ca^2+^ from intracellular reservoirs [2,36-39]. *I*_osc_ elicited by FBS were recorded from native oocytes, and those generated by ACh or ATP were recorded from oocytes expressing M1 or P2Y receptors. The *I*_osc_ amplitude was then compared among control oocytes and oocytes co-injected with as-STIM1 or as-STIM2. The results showed no significant difference among the various groups of oocytes tested for a particular agonist (Figure 3A), although average current amplitude was consistently smaller for LPAR stimulation, and larger for M1-stimulated responses. Together, these results showed that the injection of antisense oligonucleotides did not affect *I*_osc_ activation, strongly suggesting that the Ca^2+^-release mechanism remained intact and showing that its strength was dependent on the receptor type stimulated.

### Participation of STIM1 in *T*_in_ generation

To analyze the role of STIM1 and STIM2, *T*_in_ current was monitored by applying hyperpolarizing voltage steps of −90 mV every 40–60 s, from a holding potential of −10 mV (Figure 3B-E). As illustrated in Figure 3B, the *T*_in_ current amplitude generally increased after acute agonist application (either ATP, ACh, or FBS for 120 s), reached a peak after 400–480 s, and then slowly returned to basal levels after 680–800 s (65 oocytes, 12 frogs). Consistent with previous studies [6], *T*_in_ was a Cl^−^ current that was dependent on extracellular Ca^2+^, and it was blocked by lanthanides with an IC_50_ for La^3+^ of 41 ± 0.21 nM and for Gd^3+^ of 7 ± 0.23 μM, potencies similar to those shown to block SOC channels in other studies [3,6,40].

**Figure 4:**
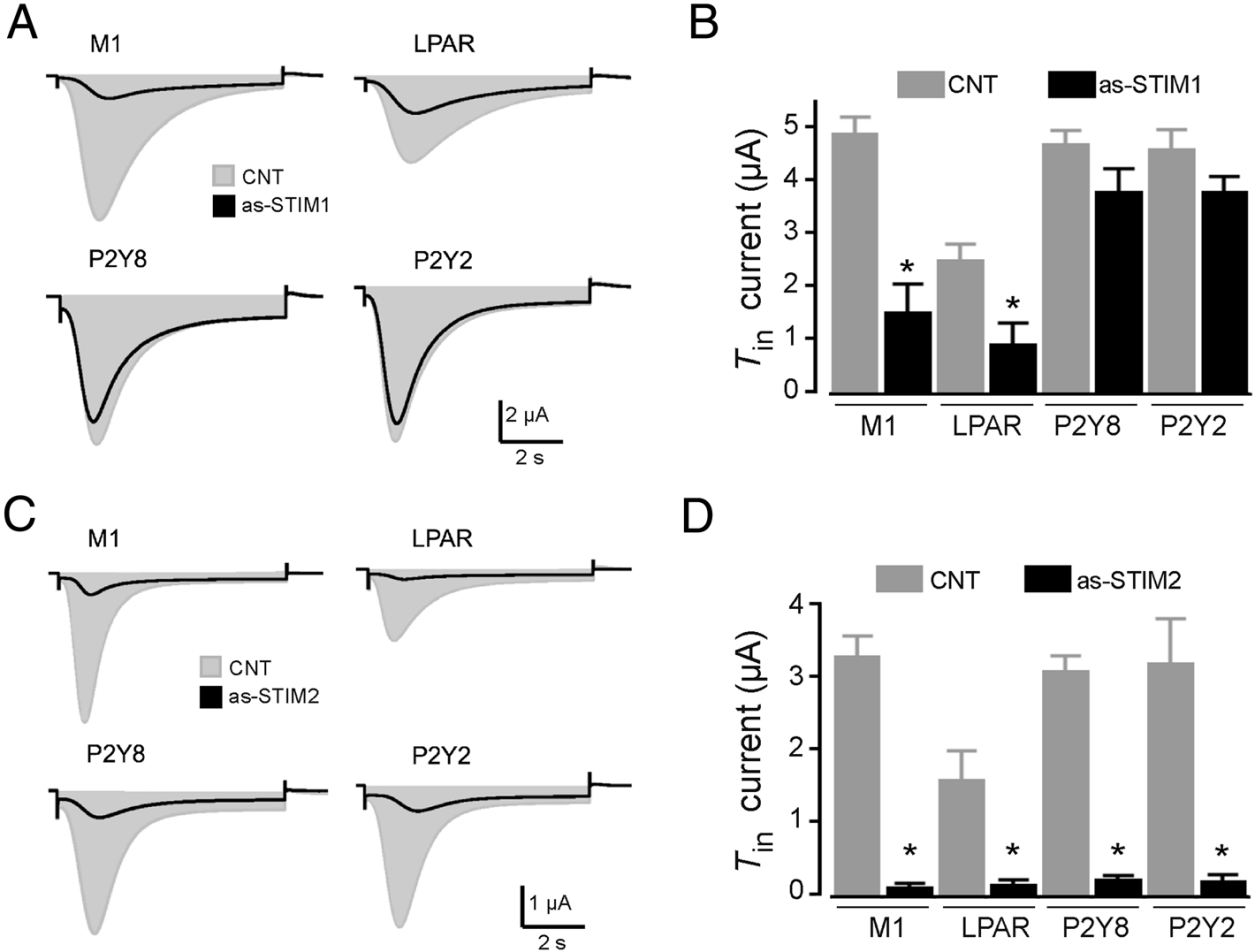
**Specific STIM knockdown by oocyte injection of as-STIM differentially decreased the** ***T***_**in**_ **current. A)** Oocytes induced to express M1, P2Y8, or P2Y2 receptors were stimulated with either ACh or ATP (100 μM), and LPAR in native oocytes were stimulated by FBS (1:1000 dilution); the resulting *T*_in_ currents (CNT, gray areas) were compared with the *T*_in_ obtained in oocytes from the corresponding group that were also injected with 50 ng as-STIM1 (superimposed black traces); all responses were monitored 48–72 h after oocyte injection. **B)** The graph shows the results obtained using the different experimental conditions illustrated in **A)**. **C)** In a set of experiments similar to those shown in **A)**, *T*_in_ currents were monitored, and the peak amplitudes of non-injected CNT oocytes were compared with those of oocytes injected (48–72 h before recording) with 50 ng as-STIM2 and stimulated with the agonists. **D)** The graph shows the results obtained using the different experimental conditions illustrated in **C)**. Bars correspond to the mean (± SEM) of the *T*_in_ peak amplitude of 10–15 oocytes from 5–6 frogs (*p < 0.01, as-STIM vs. CNT).

Then the effect of as-STIM1 injection on *T*_in_ current generation (121 oocytes, 9 frogs) was assessed. In control oocytes, application of FBS (1:1000) elicited *T*_in_ current responses of 2.5 ± 0.28 μA (Figure 4A-B). However, in as-STIM1-injected oocytes, the average *T*_in_ generated was 0.92 ± 0.38 μA, which represented a decrease of 60 ± 5.2%. Similarly, oocytes exposed to 100 μM ACh (expressing M1 receptor) showed a 70 ± 9.7% decrease in *T*_in_ in the as-STIM1-injected group. However, oocytes expressing P2Y8 and exposed to 100 μM ATP exhibited a 20 ± 1.4% decrease in *T*_in_ and oocytes expressing P2Y2 exhibited a reduction of only 15 ± 1.5% (Figure 4B). The results clearly indicated that elimination of STIM1 did not cause a complete loss of the *T*_in_ response elicited by any of the agonists used.

### STIM2 knockdown potently inhibited *T*_in_ generation regardless of the receptor stimulated

We next tested whether STIM2 knockdown affected *T*_in_ currents activated either by P2Y or M1 receptors with experiments similar to those described above. In contrast to what happened with STIM1 knockdown, as-STIM2 injection drastically reduced the *T*_in_ response elicited by the acute stimulation of any of the receptors tested (Figure 4C-D). *T*_in_ current amplitude was reduced by 96 ± 6.6% in oocytes stimulated with FBS, by 96 ± 3.6% with ACh, by 93 ± 7.1% with ATP for oocytes expressing P2Y8 receptors, and by 94 ± 8% for those expressing P2Y2. In this case, the amount of decrease observed did not differ among the receptors studied (Figure 4D). As shown above, it was evident that the decrease in the *T*_in_ response was not due to uncoupling of the IP_3_/Ca^2+^-release system since the oscillatory responses in all the oocyte groups remained unchanged.

Control experiments were also made using scrambled oligonucleotide sequences as well as as-Cx38 a different antisense oligonucleotide sequence to rule out the possibility that injection *per se* yielded nonspecific results, in these cases no effects were observed on *T*_in_ current amplitude. For example, as-Cx38 was used to knockdown connexons formed by Cx38, whose opening by superfusion of Ca^2+^-free Ringer’s solution [28,32] results in a fast and reliable test for Cx38 expression. Thus, in control oocytes the I_c_ current response was elicited by superfusion of Ca^2+^-free Ringer’s solution (3.06 ± 0.16 μA; 9 oocytes, 3 frogs) while in as-Cx38 injected oocytes the current response was eliminated. However, in the same oocytes from both groups, the Tin current amplitude was similar, regardless the membrane receptor stimulated, either M1 (2.98 ± 0.17 μA vs. 2.95 ± 0.18 μA) or P2Y8 (3.14 ± 0.14 μA vs. 3.15 ± 0.15 μA) (16 oocytes, 4 frogs).

All together, these results indicate that *T*_in_ generation in the *Xenopus* oocyte requires STIM2 protein.

### COOH-STIM2 antibody enhances the *T*_in_ current response

**Figure 5:**
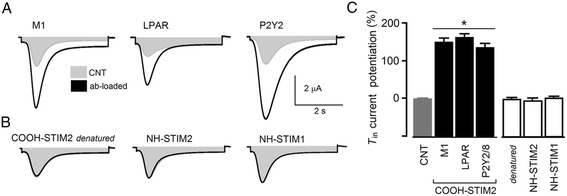
**Oocyte injection with COOH-STIM2 antibody produced a strong potentiation of** ***T***_**in**_ **current response. A)** *T*_in_ current responses were monitored in two conditions: non-loaded oocytes (CNT) and oocytes loaded with COOH-STIM2 antibody (ab-loaded). *T*_in_ responses were elicited by ACh, FBS, or ATP application, depending on the receptor to be stimulated. In all cases, a strong potentiation of the response was observed in ab-loaded oocytes. **B)** Oocytes stimulated by ACh (M1) loaded with denatured COOH-STIM2 had control-like responses, while NH-STIM2 or NH-STIM1 loading did not produce *T*_in_ potentiation. **C)** The graph shows the results obtained using the different experimental conditions illustrated in **A** and **B**; each bar corresponds to the mean (± SEM) of the *T*_in_ peak amplitude normalized against the CNT current of 10–15 oocytes from 3–6 frogs (*p < 0.01).

Envisioning that specific binding of antibody to STIM1 or STIM2 might affect the function of these proteins and then serve as a specific tool to evaluate the involvement of STIM in a particular response, we tested the same antibodies used in the Western blot for their effect on *T*_in_ generation. For this purpose, antibodies were microinjected into the oocyte cytoplasm to reach a final dilution of 1:1000. Figure 5 shows that ACh application onto oocytes pre-loaded with COOH-STIM2 resulted in a robust potentiation of the *T*_in_ response, increasing the amplitude by 158 ± 25% (15 oocytes, 5 frogs). COOH-STIM2 injection also potentiated by 168 ± 30% the *T*_in_ responses elicited by FBS, and a similar effect was observed in oocytes stimulated through the P2Y8 (126 ± 37%) or the P2Y2 receptor (129 ± 23%) (Figure 5). However, in oocytes (n = 22) from the same frogs that were injected with denatured COOH-STIM2 (incubated for 10 min at 70°C), *T*_in_ potentiation was completely abolished (Figure 5B). Also, injection of NH-STIM1 or NH-STIM2 antibody did not produce any changes in the *T*_in_ response, nor did the injection of a P2Y2 antibody.

All these results clearly indicated that the COOH-STIM2 antibody specifically potentiated the *T*_in_ current, regardless of the receptor stimulated.

### Role of STIM1 and STIM2 during long-lasting agonist stimulation

The following experiments were designed to explore the possibility that STIM1 and STIM2 have different effects, depending on the duration of the stimulus. Thus, oocytes injected with as-STIM1 or with as-STIM2 and expressing M1, P2Y8, or P2Y2 receptors were incubated for 1–4 h in the presence of their respective agonists at 1 μM (Figure 3D-E). Extended agonist incubation generated strong *T*_in_ currents that remained stable for more than the 60-min recording time, even under constant superfusion of the oocytes with NR solution, and it began to decrease after 120–180 min of wash; we assumed that in this condition the SOC machinery was over-stimulated, and that the time spent in the activated state reflected the time necessary to refill the reservoirs.

In the oocytes knocked down for STIM1, *T*_in_ currents activated by long-lasting stimulation with any of the agonists analyzed were no different from those observed in control oocytes. In contrast, in oocytes injected with as-STIM2 and expressing P2Y or M1 receptors that had been stimulated for long intervals with their respective agonists, the *T*_in_ current was no longer generated (10–15 oocytes in each group, 5 frogs), strongly suggesting that STIM2 in the oocyte was essential for responses generated through both the acute and long-lasting stimulation protocols.

### STIM proteins and the maturation process

**Figure 6:**
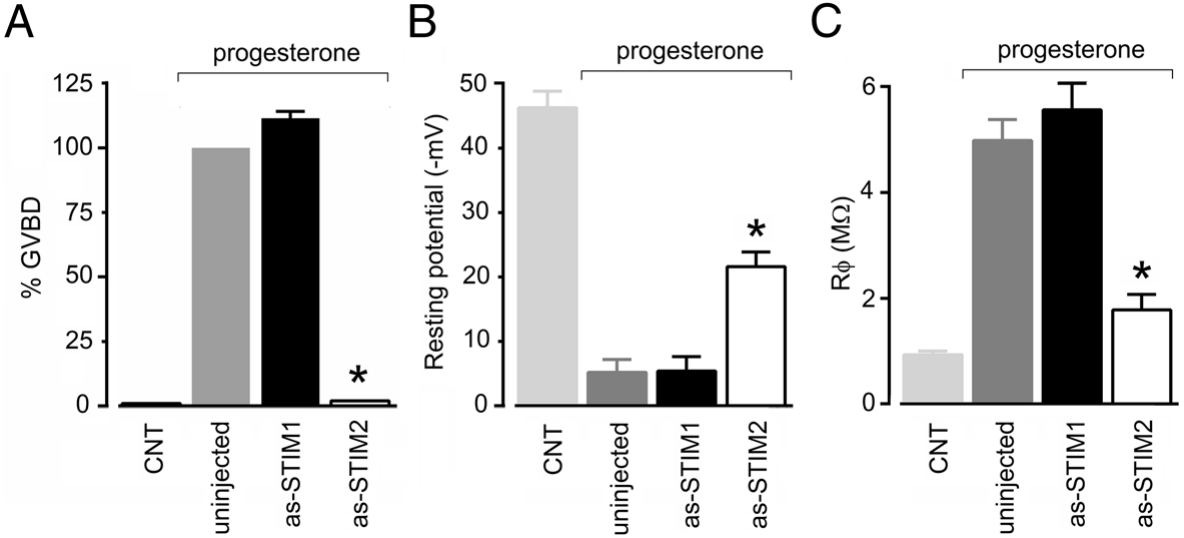
**Effect of as-STIM2 on GVBD and oocyte membrane characteristics during maturation induced by progesterone. A)** The maturation process promoted by progesterone (10 μM) was analyzed in uninjected oocytes, or in oocytes injected 72 h prior to the assay with either as-STIM1 or as-STIM2, and compared with control oocytes in the absence of progesterone. GVBD was quantified after 8–12 h in presence of progesterone (10 oocytes per group, repeated using 3 different frogs) and is normalized against the value observed in uninjected oocytes. **B)** Resting membrane potential was monitored 8–12 h after addition of progesterone in the same groups of oocytes (n = 3-5, repeated in 3 frogs) as in** A)**. **C)** The input membrane resistance (Rϕ) was estimated over the range from −80 to −20 mV in the different oocyte groups treated in the same conditions. Control groups, without progesterone, included both uninjected and antisense-injected oocytes. In all cases, values for as-STIM2-injected groups were different from as-STIM1-injected or uninjected groups (*p < 0.01).

During the maturation process, molecular elements that control the Ca^2+^ dynamics in the *Xenopus* oocyte undergo an important reconfiguration; this observation has been extended to different species, and similar changes are known to occur during mitosis [23,31,41]. Given the importance of these events for cell cycle control, we asked whether or not the knockdown of STIM proteins affected the maturation process. Thus, batches of control oocytes, and those that were injected with as-STIM1 or as-STIM2 were assayed 48–72 h after injection with 10 μM progesterone in Barth’s solution to induce maturation. Oocyte maturation entry was scored by the appearance of GVBD after 8–12 h in the presence of progesterone. The GVBD score obtained was compared against progesterone-treated control oocytes. The results are illustrated in Figure 6A; as-STIM1-injected oocytes did not show any effect on the efficiency of maturation, while STIM2 knockdown produced a strong inhibition of the process (the experiment was repeated in oocytes from 3 different donors). Lack of GVBD in as-STIM2-injected oocytes seemed to indicate a failure to enter meiosis and a consequent incomplete maturation process; this interpretation was also supported by monitoring electrophysiological parameters in all of the groups tested. As illustrated in Figure 6B-C, electrical parameters of as-STIM2-injected oocytes (progesterone-treated) were different from those displayed by as-STIM1-injected oocytes and control oocytes maintained in progesterone for the same period of time.

Taken together, these results clearly showed that in oocytes where STIM2 was knocked down, the process of maturation was inhibited at some early point. The first manifestation of this was the complete blockage of GVBD, i.e., of the signal for meiosis entry; this result was clearly different from that observed in STIM1-deprived oocytes.

## Discussion

Here, using biochemical strategies and electrophysiology, we studied what effects the knockdown of endogenous STIM proteins had on two important *Xenopus* oocyte responses: the activation of SOCE monitored by measuring *T*_in_ current generation, and the maturation process induced by progesterone. We found that: *i)* Both STIM1 and STIM2 proteins were endogenously expressed in the *Xenopus* oocyte; *ii)* Injection of antisense oligonucleotide sequences of STIM1 or STIM2 potently knocked down the expression of both the corresponding mRNA and the protein; *iii)* STIM1 or STIM2 knockdown did not seem to affect the Ca^2+^-signaling machinery responsible for generating oscillatory Ca^2+^-signals in the oocyte; *iv)* STIM2, but not STIM1, proved to be fundamental for *T*_in_ current generation; this was observed both in acute stimulation protocols or after long-lasting stimulation periods, and it did not depend on the receptor type stimulated; *v)* STIM2 protein knockdown blocked entry into the process of maturation induced by progesterone, while STIM1 elimination did not affect this process; and *vi*) an antibody against the COOH terminus of STIM2 potentiated *T*_in_ current generation.

Calcium release and influx are two phenomena well studied in the *Xenopus* oocyte. The main subject addressed here is the identity and role of STIM proteins during calcium influx stimulated through endogenous responses. It is known that after GPCR stimulation, both endogenous as well as exogenously expressed GPCR generate in the oocyte mainly two Ca^2+^-dependent Cl^−^ ion currents, one due to intracellular Ca^2+^ release that is normally followed by another current dependent on Ca^2+^ influx; this pattern is generated through an enzymatic cascade involving IP_3_ synthesis, a common mechanism in most cell systems [1]. Following the original nomenclature, in the oocyte the first response is named *I*_osc_, while the second generates the *T*_in_ current response [6]. The Ca^2+^-influx magnitude is directly related to the amplitude of Ca^2+^-release; the main molecular element responsible for this linkage is the STIM protein, since it is the Ca^2+^-sensor within the Ca^2+^ reservoir. As in previous studies [8,42,43], to monitor the [Ca^2+^]_i_ increase produced by both mechanisms in the oocyte, here we used the Ca^2+^-dependent Cl^−^ current as an endogenous sensor whose amplitude accurately reflects the concentration of Ca^2+^ beneath the plasmatic membrane. This is especially true for Ca^2+^ influx, since this occurs in the plasma membrane where the Ca^2+^-dependent Cl^−^ channels are co-expressed with the SOC channels responsible for the influx. Thus, monitoring an endogenous Ca^2+^ sensor such as the Cl^−^ channel offers not only spatial and temporal advantages, but also amplifies the normally small Ca^2+^ current through SOC channels and avoids altering the Ca^2+^ dynamics with further pharmacological manipulations. STIM protein expression and function was then studied in the *Xenopus* oocyte using this tool.

It is well known that when Ca^2+^ is released from the ER, STIM proteins are activated, rapidly translocated, and oligomerized into junctions formed between the ER and the plasma membrane, where they bind to and activate highly selective Ca^2+^ channels formed by Orai proteins that allow Ca^2+^ influx [44,45]. The main trigger for this phenomenon is a decrease in ER Ca^2+^ content; however, evidence indicates that isoforms of STIM2 protein might maintain a basal activation of Orai channels without prior Ca^2+^ release, thereby controlling the cytoplasmic Ca^2+^ concentration [12,46]. The ratio of STIM1 to STIM2 expression seems to depend on the cell type, and perhaps on the pathophysiological state; as shown here for the *Xenopus* oocyte, other cells such as T cells, myoblasts, skeletal muscle, and liver cells also co-express both STIM proteins [20,47-49]. Studies to distinguish the roles of STIM1 and STIM2 in various cells have employed diverse silencing strategies and overexpression. The injection of an antisense oligonucleotide sequence for each STIM protein was chosen here for its simplicity and because it excludes potential non-specific effects caused by protein over-expression. The effect of antisense injection on STIM expression was demonstrated by analyzing both its mRNA and protein expression by RT-PCR and Western blot, respectively. This analysis demonstrated that expression of STIM1 or STIM2 was strongly downregulated in oocytes injected with the corresponding antisense oligonucleotide sequence. This antisense effect was not affected by co-expression of GPCR proteins, used experimentally to stimulate Ca^2+^-release. Also, it was shown that STIM protein knockdown did not affect the IP_3_ increase and subsequent Ca^2+^ release, as indicated by the *I*_osc_ amplitude responses evoked at the beginning of each experiment by acute application of one of the agonists studied. In addition, preliminary results using RT-PCR showed amplification of transcripts for Orai1 and Orai2 in the oocyte (not shown).

Oocytes injected with either antisense STIM1 or STIM2 were then monitored to analyze their ability to generate *T*_in_ current using two stimulation protocols. In the first, acute application of the agonist produced the typical *I*_osc_ response; in control conditions it was followed by *T*_in_ generation that declined after 680–800 s. In the second protocol, long-lasting (1–4 h) stimulation of control oocytes with a low concentration of agonist (1 μM) gave strong *T*_in_ current responses that remained active for 60–180 min, even in constant superfusion with NR solution. A possible explanation for this difference in response kinetics is that prolonged stimulation produced a stronger activation of the SOCE mechanism, probably due to a more marked decrease in ER Ca^2+^ concentration. Both protocols were applied in oocytes in which STIM1 or STIM2 expression had been eliminated. The STIM2 knockdown produced a severe decrease in *T*_in_ current generation (93 - 100%) in both stimulation protocols, indicating that STIM2 was indispensable to induce the *T*_in_ current response. Using acute receptor stimulation, elimination of STIM1 caused a smaller but significant decrease in *T*_in_ current generation thus, STIM2 alone was unable to support full *T*_in_ activation during acute stimulation, suggesting that an association of STIM2 with STIM1 was necessary in order to activate the endogenous response. Also it was observed that STIM1 requirement seemed to be minor during P2Y purinergic stimulation, this difference cannot be explained by the amplitudes of *I*_osc_ generated by the agonists, given that ACh and LPA were the more and less effective, respectively, in generating the response, and both agonists showed similar patterns of *T*_in_ decrease by STIM1 knockdown. Thus, this result might indicate some type of molecular specificity, perhaps intrinsic to the molecules involved or as a consequence of their differential expression and localization in the oocyte membrane. Differential insertion of several proteins expressed in the oocyte membrane has been demonstrated; thus, membrane domains with greater expression of STIM2 together with P2Y receptors are plausible.

A central role for STIM2 protein in SOCE generation has been shown before in some cell types such as neurons [20,50] and dendritic cells [19]; however, there is no information indicating whether or not the expression of STIM1 might affect the full endogenous response in these cases. For example, it has been shown the essential role of STIM2 in SOCE activation in dendritic spines [50], which was not substituted by overexpression of STIM1. The authors concluded that this is due to differences in STIM-Ca^2+^ sensitivity and subcellular localization of the proteins. In many other cell systems, co-participation or complementary roles for the two STIM proteins have been postulated [15,48]. Finally, when the oocytes were stimulated using the long-lasting protocol, elimination of STIM1 had no effect indicating that, in this case, the *T*_in_ current was activated by STIM2 alone. The central role for STIM2 is supported by the latter result as well as by the finding that injecting the antibody (COOH-STIM2) against STIM2 specifically increased the current amplitude by more than 100%, regardless of the agonist used to generate the *T*_in_ response. It is known that the COOH region, in both STIM1 and STIM2, contains the domain necessary to interact and activate the SOC channel formed by Orai [44]. Thus, a potentiating effect of the COOH-STIM2 antibody indicates that the strength of the STIM2-Orai interaction might be regulated, either positively or negatively, through a site that is affected by the antibody, indirectly confirming the central role of STIM2 during *T*_in_ generation. As expected given the STIM structure proposed, the two antibodies that recognized domains close to the amino-terminus had no effect on the *T*_in_ response.

Significant inhibition of the maturation process was observed in oocytes devoid of STIM2 protein. Here, we provided clear-cut evidence of STIM2 involvement during or in preparation for maturation, since its absence eliminated the process of GVBD. Once again, this result contrasted with the lack of effect in STIM1-knockdown oocytes, whose maturation was similar to that of control oocytes. Indeed, it has been shown that the function of STIM1 is downregulated during the maturation process, which contributes to elimination of the SOC response in *Xenopus* oocytes [22-24]; a similar condition has been shown in the mammalian oocyte [25], although in the latter this phenomenon remains controversial [51]. There is no previous information regarding the effect produced by lack (or overexpression) of STIM2 during maturation either in frog or mammalian oocytes, as most previous studies focused on the role of STIM1. However, mouse oocyte is known to express STIM2 protein in the ER; during maturation, STIM2 re-localizes from a homogeneous distribution to one closer to the meiotic spindles, suggesting a role during this process [52]. In frog, is possible that the inhibitory effect of STIM2 knockdown was unrelated to its role in SOCE activation, since meiosis entry in *Xenopus* does not require Ca^2+^-influx [42]. Further studies will be necessary to characterize the level at which the lack of STIM2 had such a dramatic effect on the maturation process, and to determine if it might have more general implications. One possibility is that as-STIM2 might cause downregulation of a STIM2 isoform different from that involved in SOCE activation. Another possibility relates to its role regulating cytoplasmic [Ca^2+^], in which case as-STIM2 might affect the activation of Ca^2+^-dependent processes required prior to meiosis entry.

## Conclusion

In this study, STIM2 is fundamental for the endogenous SOC response in the oocyte, although an association with STIM1 seemed to be necessary for its full generation. The mechanism responsible for the clear dependence of meiosis entry on STIM2 expression is a fundamental question that remains open, and its elucidation might help to understand the function of STIM proteins in the *Xenopus* oocyte and in other cell types as well.

## Additional file

Additional file 1:
**BLAST analysis for the amplified sequences of stim1 and stim2.** Alignment produced by BLAST for the amplified sequences of stim1 (stim1X1a) (Panel A) and stim2 (stim2X1) (Panel B) with the sequences reported for stim1X1 (GenBank accession number NM_001097037.1) and stim2Xt (GenBank accession number XM_004916759.1), respectively.

## References

[1] Berridge MJ, Lipp P, Bootman MD (2000). The versatility and universality of calcium signalling. Nat Rev Mol Cell Biol.

[2] Parker I, Miledi R (1986). Changes in intracellular calcium and in membrane currents evoked by injection of inositol trisphosphate into *Xenopus* oocytes. Proc R Soc Lond B Biol Sci.

[3] Yao Y, Tsien R (1997). Calcium current activated by depletion of calcium stores in *Xenopus* oocytes. J Gen Physiol.

[4] Hartzell HC: **Activation of different Cl**^**−**^**currents in Xenopus oocytes by Ca**^**2+**^**liberated from stores and by capacitative Ca**^**2+**^**influx.** *J Gen Physiol* 1996, **108:**157–175. 10.1085/jgp.108.3.157 PMC2229319 8882861

[5] Parekh AB, Penner R (1997). Store depletion and calcium influx. Physiol Rev.

[6] Parker I, Gundersen C, Miledi R (1985). A transient inward current elicited by hyperpolarization during serotonin activation in *Xenopus* oocytes. Proc R Soc Lond B Biol Sci.

[7] Sun L, Machaca K: **Ca**^**2+**^**(cyt) negatively regulates the initiation of oocyte maturation.** *J Cell Biol* 2004, **165:**63–75. 10.1083/jcb.200309138 PMC1289150 15067021

[8] Williams RT, Manji SS, Parker NJ, Hancock MS, Van Stekelenburg L, Eid JP, Senior PV, Kanzenwadel JS, Shandala T, Saint R, Smith PJ, Dziadek MA (2001). Identification and characterization of the STIM (stromal interaction molecule) gene family: coding for a novel class of transmembrane proteins. Biochem J.

[9] Roos J, DiGregorio PJ, Yeromin AV, Ohlsen K, Lioudyno M, Zhang S, Safrina O, Kozak JA, Wagner SL, Cahalan MD, Veliçelebi G, Stauderman KA: **STIM1, an essential and conserved component of store-operated Ca**^**2+**^**channel function.** *J Cell Biol* 2005, **169:**435–445. 10.1083/jcb.200502019 PMC2171946 15866891

[10] Klein SL, Strausberg RL, Wagner L, Pontius J, Cllifton SW, Richardson P (2002). Genetic and genomic tools for *Xenopus* research: The NIH *Xenopus* initiative. Dev Dyn.

[11] Soboloff J, Rothberg BS, Madesh M, Gill DL (2012). STIM proteins: dynamic calcium signal transducers. Nat Rev Mol Cell Biol.

[12] Brandman O, Liou J, Park WS, Meyer T: **STIM2 is a feedback regulator that stabilizes basal cytosolic and endoplasmic reticulum Ca**^**2+**^**levels.** *Cell* 2007, **131:**1327–1339. 10.1016/j.cell.2007.11.039 PMC2680164 18160041

[13] Frischauf I, Schindl R, Derler I, Bergsmann J, Fahrner M, Romanin C (2008). The STIM/Orai coupling machinery. Channels (Austin).

[14] López E, Salido GM, Rosado JA, Berna-Erro A (2012). Unraveling STIM2 function. J Physiol Biochem.

[15] Gruszczynska-Biegala J, Pomorski P, Wisniewska MB, Kuznicki J (2011). Differential roles for STIM1 and STIM2 in store-operated calcium entry in rat neurons. PLoS One.

[16] Stiber J, Hawkins A, Zhang ZS, Wang S, Burch J, Graham V, Ward CC, Seth M, Finch E, Malouf N, Williams RS, Eu JP, Rosenberg P (2008). STIM1 signalling controls store-operated calcium entry required for development and contractile function in skeletal muscle. Nat Cell Biol.

[17] Varga-Szabo D, Authi KS, Braun A, Bender M, Ambily A, Hassock SR, Gudermann T, Dietrich A, Nieswandt B: **Store-operated Ca**^**2+**^**entry in platelets occurs independently of transient receptor potential (TRP) C1.** *Pflugers Arch* 2008, **457:**377–387. 10.1007/s00424-008-0531-4 18546016

[18] Zhang SL, Yu Y, Roos J, Kozak JA, Deerinck TJ, Ellisman MH, Stauderman KA, Cahalan MD: **STIM1 is a Ca**^**2+**^**sensor that activates CRAC channels and migrates from the Ca**^**2+**^**store to the plasma membrane.** *Nature* 2005, **437:**902–905. 10.1038/nature04147 PMC1618826 16208375

[19] Bandyopadhyay BC, Pingle SC, Ahern GP: **Store-operated Ca**^**2+**^**signaling in dendritic cells occurs independently of STIM1.** *J Leukoc Biol* 2010, **89:**57–62. 10.1189/jlb.0610381 PMC3004519 20971921

[20] Berna-Erro A, Braun A, Kraft R, Kleinschnitz C, Schuhmann MK, Stegner D, Wultsch T, Eilers J, Meuth SG, Stoll G, Nieswandt B: **STIM2 regulates capacitive Ca**^**2+**^**entry in neurons and plays a key role in hypoxic neuronal cell death.** *Sci Signal* 2009, **2:**ra67. 10.1126/scisignal.2000522 19843959

[21] Song MY, Makino A, Yuan JX-J: **STIM2 Contributes to enhanced store-operated Ca**^**2+**^**entry in pulmonary artery smooth muscle cells from patients with idiopathic pulmonary arterial hypertension.** *Pulm Circ* 2011, **1:**84–94. 10.4103/2045-8932.78106 PMC3121304 21709766

[22] El-Jouni W, Jang B, Haun S, Machaca K (2005). Calcium signaling differentiation during *Xenopus* oocyte maturation. Dev Biol.

[23] Machaca K, Haun S: **Induction of maturation-promoting factor during*****Xenopus*****oocyte maturation uncouples Ca**^**2+**^**store depletion from store-operated Ca**^**2+**^**entry.** *J Cell Biol* 2002, **156:**75–85. 10.1083/jcb.200110059 PMC1307503 11781335

[24] Yu F, Sun L, Machaca K (2009). Orai1 internalization and STIM1 clustering inhibition modulate SOCE inactivation during meiosis. Proc Natl Acad Sci U S A.

[25] Cheon B, Lee H-C, Wakai T, Fissore RA: **Ca**^**2+**^**influx and the store-operated Ca**^**2+**^**entry pathway undergo regulation during mouse oocyte maturation.** *Mol Biol Cell* 2013, **24:**1396–1410. 10.1091/mbc.E13-01-0065 PMC3639051 23468522

[26] Abdullaev IF, Bisaillon JM, Potier M, Gonzalez JC, Motiani RK, Trebak M (2008). Stim1 and Orai1 mediate CRAC currents and store-operated calcium entry important for endothelial cell proliferation. Circ Res.

[27] El Boustany C, Katsogiannou M, Delcourt P, Dewailly E, Prevarskaya N, Bprpwiec AS, Capiod T (2010). Differential roles of STIM1, STIM2 and Orai1 in the control of cell proliferation and SOCE amplitude in HEK293 cells. Cell Calcium.

[28] Arellano RO, Robles-Martínez L, Serrano-Flores B, Vázquez-Cuevas F, Garay E: **Agonist-activated Ca**^**2+**^**influx and Ca**^**2+**^**-dependent Cl**^**−**^**channels in*****Xenopus*****ovarian follicular cells: functional heterogeneity within the cell monolayer.** *J Cell Physiol* 2012, **227:**3457–3470. 10.1002/jcp.24046 22213197

[29] Dumont JN (1972). Oogenesis in *Xenopus laevis* (Daudin). I. Stages of oocyte development in laboratory maintained animals. J Morphol.

[30] Hulstrand AM, Schneider PN, Houston DW (2010). The use of antisense oligonucleotides in *Xenopus* oocytes. Methods.

[31] Saldaña C, Garay E, Rangel GE, Reyes LM, Arellano RO (2009). Native ion current coupled to purinergic activation via basal and mechanically induced ATP release in *Xenopus* follicles. J Cell Physiol.

[32] Arellano RO, Woodward RM, Miledi R (1995). A monovalent cationic conductance that is blocked by extracellular divalent cations in Xenopus oocytes. J Physiol.

[33] Galán C, Zbidi H, Bartegi A, Salido GM, Rosado JA (2009). STIM1, Orai1 and hTRPC1 are important for thrombin- and ADP-induced aggregation in human platelets. Arch Biochem Biophys.

[34] López JJ, Salido GM, Pariente JA, Rosado JA: **Interaction of STIM1 with endogenously expressed human canonical TRP1 upon depletion of intracellular Ca**^**2+**^**stores.** *J Biol Chem* 2006, **281:**28254–28264. 10.1074/jbc.M604272200 16870612

[35] Tigyi G, Dyer D, Matute C, Miledi R (1990). A serum factor that activates the phosphatidylinositol phosphate signaling system in *Xenopus* oocytes. Proc Natl Acad Sci U S A.

[36] Miledi R, Parker I (1984). Chloride current induced by injection of calcium into *Xenopus* oocytes. J Physiol.

[37] Nomura Y, Kaneko S, Kato K, Yamagishi S, Sugiyama H (1987). Inositol phosphate formation and chloride current responses induced by acetylcholine and serotonin through GTP-binding proteins in *Xenopus* oocyte after injection of rat brain messenger RNA. Brain Res.

[38] Oron Y, Dascal N, Nadler E, Lupu M (1985). I**nositol 1,4,5-trisphosphate mimics muscarinic response in*****Xenopus*****oocytes**. Nature.

[39] Takahashi T, Neher E, Sakmann B (1987). Rat brain serotonin receptors in Xenopus oocytes are coupled by intracellular calcium to endogenous channels. Proc Natl Acad Sci U S A.

[40] Putney JW (2010). Pharmacology of store-operated calcium channels. Mol Interv.

[41] Preston SF, Sha’afi RI, Berlin RD: **Regulation of Ca**^**2+**^**influx during mitosis: Ca**^**2+**^**influx and depletion of intracellular Ca2+ stores are coupled in interphase but not mitosis.** *Cell Regul* 1991, **2:**915–925. 10.1091/mbc.2.11.915 PMC361890 1809398

[42] Petersen CC, Berridge MJ (1994). The regulation of capacitative calcium entry by calcium and protein kinase C in *Xenopus* oocytes. J Biol Chem.

[43] Parker I, Ivorra I (1992). Characteristics of membrane currents evoked by photoreleased inositol trisphosphate in *Xenopus* oocytes. Am J Physiol.

[44] Cahalan MD: **STIMulating store-operated Ca**^**2+**^**entry.** *Nat Cell Biol* 2009, **11:**669–677. 10.1038/ncb0609-669 PMC2721799 19488056

[45] Rothberg BS, Wang Y, Gill DL (2013). **Orai channel pore properties and gating by STIM: implications from the Orai crystal structure**. Sci Signal.

[46] Hoth M, Niemeyer BA (2013). The neglected CRAC proteins: Orai2, Orai3, and STIM2. Curr Top Membr.

[47] Oh-Hora M, Yamashita M, Hogan PG, Sharma S, Lamperti E, Chung W, Prakriya M, Feske S, Rao A (2008). Dual functions for the endoplasmic reticulum calcium sensors STIM1 and STIM2 in T cell activation and tolerance. Nat Immunol.

[48] Kar P, Bakowski D, Di Capite J, Nelson C, Parekh AB: **Different agonists recruit different stromal interaction molecule proteins to support cytoplasmic Ca**^**2+**^**oscillations and gene expression.** *Proc Natl Acad Sci U S A* 2012, **109:**6969–6974. 10.1073/pnas.1201204109 PMC3344999 22509043

[49] Darbellay B, Arnaudeau S, Ceroni D, Bader C, Koning S, Bernheim L (2010). **Human muscle economy myoblast differentiation and excitation-contraction coupling use the same molecular partners, STIM1 and STIM2**. J Biol Chem.

[50] Sun S, Zhang H, Liu J, Popugaeva E, Xu NJ, Feske S, White CL, Bezprozvanny I (2014). Reduced synaptic STIM2 expression and impaired store-operated calcium entry cause destabilization of mature spines in mutant presenilin mice. Neuron..

[51] Gomez-Fernandez C, Lopez-Guerrero AM, Pozo-Guisado E, Álvarez IS, Matín-Romero FJ (2012). **Calcium signaling in mouse oocyte maturation: the roles of STIM1, ORAI1 and SOCE**. Mol Hum Reprod.

[52] Miao Y-L, Williams CJ (2012). Calcium signaling in mammalian egg activation and embryo development: the influence of subcellular localization. Mol Reprod Dev.

