# Supplementary material for: Differential role of STIM1 and STIM2 during transient inward (Tin) current generation and the maturation process in the Xenopus oocyte
Source: BMC Physiol. 2014 Nov 15;14:9. doi: 10.1186/s12899-014-0009-x (PMC4236480; doi:10.1186/s12899-014-0009-x)
Supplement: Additional file 1: — BLAST analysis for the amplified sequences of stim1 and stim2. Alignment produced by BLAST for the amplified sequences of stim1 (stim1X1a) (Panel A) and stim2 (stim2X1) (Panel B) with the sequences reported for stim1X1 (GenBank accession number NM_001097037.1) and stim2Xt (GenBank accession number XM_004916759.1), respectively. [file 12899_2014_9_MOESM1_ESM.pdf]

Panel A

```
stim1Xla  1     CCCACAGCCAAGCACAGCACCTTCCATGGAGAGGACAAGCTGATCAGTGTGGAGGATCTT  60
                ||||||||||||||||||||||||||||||||||||||||||||||||||||||||||||
stim1Xl   369   CCCACAGCCAAGCACAGCACCTTCCATGGAGAGGACAAGCTGATCAGTGTGGAGGATCTT  428

stim1Xla  61    TGGAACAGCTGGAAAATATCAGAAGTCTATAACTGGACGGTGGATGAAGTGGCCCAGTGG  120
                ||||||||||||||||||||||||||||||||||||||||||||||||||||||||||||
stim1Xl   429   TGGAACAGCTGGAAAATATCAGAAGTCTATAACTGGACGGTGGATGAAGTGGCCCAGTGG  488

stim1Xla  121   TTGATCACATATGTGGAGCTGCCCCAGTATGAAGAGACATTCCGCAAGCTGCAGCTCAGT  180
                ||||||||||||||||||||||||||||||||||||||||||||||||||||||||||||
stim1Xl   489   TTGATCACATATGTGGAGCTGCCCCAGTATGAAGAGACATTCCGCAAGCTGCAGCTCAGT  548

stim1Xla  181   GGCAGAGACATGCCCAGGTTGGCAATCGCCAATGCCACCATGACGGGCACCCTCCTGAAA  240
                |||||||||||||||||||||||||||||||| |||||||||||||||||||||||||||
stim1Xl   549   GGCAGAGACATGCCCAGGTTGGCAATCGCCAACGCCACCATGACGGGCACCCTCCTGAAA  608

stim1Xla  241   ATGACCGACCGCAGTCAGAGGCAGAAGCTGCAGCTCAAGGCGTTGGACACGGTGCTGTTT  300
                ||||||||||||||||||||||||||||||||||||||||||||||||||||||||||||
stim1Xl   609   ATGACCGACCGCAGTCAGAGGCAGAAGCTGCAGCTCAAGGCGTTGGACACGGTGCTGTTT  668

stim1Xla  301   GGGCCTCCTCTGTTGACTCGTCACAATCATCTCAAGGA  338
                ||||||||||||||||||||||||||||||||||||||
stim1Xl   669   GGGCCTCCTCTGTTGACTCGTCACAATCATCTCAAGGA  706
```

Panel B

```
stim2Xl  1      CCATTGGCACAGAGAAGATAAGCATATCACAGTTGAAGATCTGTGGGCGCAATGGAAAAC  60
                ||||| ||||||||||||||| ||||| ||||||||||||||||||||||||||||||||
stim2Xt  715    CCATTTGCACAGAGAAGATAAACATATAACAGTTGAAGATCTGTGGGCGCAATGGAAAAC  774

stim2Xl  61     CTCTGAAGCTCACAACTGGACCGAAGAGGAAACGTTGCAGTGGTTGCTGGAATTTGTAGA  120
                |||||||| ||| |||||||| |||||||| || ||||||||||||||||||||||| ||
stim2Xt  775    CTCTGAAGTTCATAACTGGACTGAAGAGGAGACTTTGCAGTGGTTGCTGGAATTTGTGGA  834

stim2Xl  121    GCTCCCTCAGTATGAGAAGACCTTTAGAGAGAATTCTGTGAAAGGGACAACTTTGCCCAG  180
                ||| |||||||||||||||| |||||||||||||||||| ||||| ||| || |||| ||
stim2Xt  835    GCTTCCTCAGTATGAGAAGAACTTTAGAGAGAATTCTGTCAAAGGAACAGCTCTGCCAAG  894

stim2Xl  181    GATAGCTGTTAATGAGCCCCCATTAATGATCTCGCATCTTAAAATTACTGATCGGAGCCA  240
                |||||||||||||||||     |||||||||| || ||||||||||| |||||||||||
stim2Xt  895    AATAGCTGTTAATGAGCCTGTGCTAATGATCTCACAACTTAAAATTACCGATCGGAGCCA  954

stim2Xl  241    CAGGCAAAAGCTTCAGCTGAAAGCGATGGACGTTGTGTTGTTCGGGCCACCAACAAGACC  300
                |||||||||  |||||||||||||  ||||||| ||||| ||||||||||||||||||||
stim2Xt  955    CAGGCAAAAATTTCAGCTGAAAGCATTGGACGTAGTGTTATTCGGGCCACCAACAAGACC  1014

stim2Xl  301    TCCGCACAACTGGGTTAAGGATTTTATCCTGACAATTTCTATTGTAATCGGAGTTGGAGG  360
                || ||||||||||| || |||||||||||||| ||||||||||||||||||||||||||
stim2Xt  1015   ACCACACAACTGGGTAAAAGATTTTATCCTGACCATTTCTATTGTAATCGGAGTTGGAGG  1074

stim2Xl  361    TTG  363
                |||
stim2Xt  1075   TTG  1077
```
